# Opportunities and challenges of trilateral South‒South cooperation for transforming development assistance for health: evidence from a DRC–UNICEF–China maternal, newborn, and child health project

**DOI:** 10.1186/s12992-023-00934-9

**Published:** 2023-06-08

**Authors:** Aidan Huang, Chunkai Cao, Angela Y. Xiao, Hermès Karemere, Molima E. Christian, Kenanewabo K. Nicolas, Meng Xue, Kun Tang

**Affiliations:** 1 Vanke School of Public Health, Tsinghua University, No. 30 Shuangqing Road, Beijing, 100084 China; 2 Institute for International and Area Studies, Tsinghua University, No. 30 Shuangqing Road, Beijing, 100084 China; 3 École Régionale de Santé Publique, Université Catholique de Bukavu (UCB), Avenue Michombero n°2, Kadutu, Bukavu, Democratic Republic of Congo; 4 Division Provinciale de la Santé (DPS), du Kasai Central, Democratic Republic of Congo

**Keywords:** Trilateral cooperation, South-South cooperation, Development assistance for health, Maternal, newborn, and child health, Democratic Republic of Congo, United Nations Children’s Fund, China

## Abstract

**Background:**

Trilateral South‒South cooperation is envisioned as an equal and empowering partnership model but still faces certain challenges. This study addresses whether and how trilateral South‒South cooperation can transform traditional development assistance for health (DAH) and explores the opportunities and challenges of trilateral South‒South cooperation for transforming future DAH, in the theme of “the emerging development partner’s DAH transformation facilitated by a multilateral organization”.

**Methods:**

We evaluate a maternal, newborn, and child health (MNCH) project involving the Democratic Republic of Congo (DRC), the United Nations Children’s Fund (UNICEF), and China (hereinafter referred to as the “DRC–UNICEF–China project”). We analyze data from project documents and seventeen semi-structured interviews using a pragmatic analytical framework based on the DAH program logic model and the OECD’s trilateral cooperation framework.

**Results:**

Evidence from the DRC–UNICEF–China MNCH project suggests that trilateral South‒South cooperation facilitated by a multilateral organization can provide transformative opportunities for emerging development partners’ DAH to generate and deliver context-based, demand-oriented solutions, harmonize rules and procedures, institutionalize mutual learning and knowledge sharing, and increase the visibility of emerging development partners as sources for South‒South development experience transfer. However, the project revealed some challenges, including the neglect of key stakeholders in the complex governance structure, the high transaction costs needed to ensure transparency, and the harm local absence of the emerging development partner poses to long-term DAH engagement.

**Conclusions:**

This study echoes some of the findings in trilateral SSC literature that claim power structures and philanthropic, normative justification for health equity are often juxtaposed in trilateral SSC partnerships. The opportunities offered by the DRC–UNICEF–China project align with China’s cognitive learning process for strengthening international engagement and global image building. However, challenges may arise as a result of complex governance structures and the entrustment of facilitating partners, which can threaten the effectiveness of trilateral cooperation. We call for strengthening the beneficiary partner’s ownership at all levels, engaging the emerging development partner to better understand the beneficiary partner’s local context(s) and needs, and ensuring available resources to support programmatic activities and long-term partnerships for the health and well-being of the beneficiaries.

**Supplementary Information:**

The online version contains supplementary material available at 10.1186/s12992-023-00934-9.

## Background

Trilateral South‒South cooperation (or “South‒South and triangular cooperation”) offers opportunities to transform development assistance for health (DAH) in the changing development cooperation landscape [1]. Compared with the bilateral model, trilateral cooperation is envisioned as an opportunity to embrace greater inclusivity and horizontality [2–4], entail more value added through knowledge sharing and mutual learning [5], and foster stronger and more trusting partnerships, which may extend beyond specific projects and facilitate aid coordination [6–8]. The involvement of emerging development partners can infuse the principles of South‒South cooperation (SSC), such as equality, non-interference, and non-conditionality [9], into trilateral cooperation [10, 11]. Thus, it can potentially redress the major shortcomings of North-South DAH, namely donor‒recipient hierarchy, vertical decision-making structures, and aid conditionality [1, 12]. Furthermore, trilateral partnerships can address common pitfalls of SSC, such as poor coordination, language barriers, and inadequate financing opportunities [13].

Nevertheless, trilateral SSC has been considered a subsidiary in DAH [1, 14, 15]. Contrary to the abovementioned transformative role it could play, some critics have depicted trilateral SSC as “massaging consent” [14], co-opting emerging development partners into existing hegemonies of development ideology and practices [14, 16–18], and thus reinforcing “the traditional North–South hierarchy” [1]. Challenges such as increased coordination difficulties, high transaction costs, long negotiation processes, and unclear divisions of roles and responsibilities, among others, have also been documented. [6, 19]. Moreover, the cost‒benefit calculations and necessary conditions enabling trilateral cooperation to deliver effective results continue to be debated internationally [1, 6, 14, 20].

This study aims to contribute to this debate by exploring: does trilateral SSC transform traditional DAH and, if so, how? What are the opportunities and challenges of trilateral SSC for transforming future DAH? While trilateral cooperation has received much scholarly attention, only a few studies on trilateral cooperation and SSC have empirically examined the transformative role trilateral cooperation can play in development assistance [1, 21–23]. Much of the current literature has provided generic descriptive mappings and summaries of existing projects, or conceptualized the motivations and prospects of development partners for engaging in trilateral cooperation (see Additional File 1 for a review of the literature on South‒South and trilateral cooperation). To address this gap, we conducted a case study on a maternal, newborn, and child health (MNCH) project involving the Democratic Republic of Congo (DRC), the United Nations Children’s Fund (UNICEF), and China (hereinafter referred to as the “DRC–UNICEF–China project”).

In the past, much of China’s DAH has typically been bilateral and piecemeal [24], largely packaged with Chinese overseas investments and trade deals [25], such as the dispatch of the Chinese medical teams (CMTs), construction of health facilities, and donation of essential drugs and medical equipment [26, 27]. However, widespread international concern has emerged over the effectiveness and sustainability of China’s aid [28–30], as well as domestic criticism over the value of foreign aid [31]. In the last decade, China has embarked on a path to transform its foreign aid system to improve aid effectiveness [26, 32, 33]. As such, the country has increasingly embraced trilateral cooperation with a broader range of development partners. Yet, China has also continued to defend the uniqueness of its aid vis-à-vis the West, reaffirming SSC principles as fundamental to its DAH strategy [21]. This conundrum stems from China’s ambition to engage more actively in global health governance and its pragmatic considerations to learn selectively from the West to improve its aid delivery [21, 34]. However, critics claim that China’s practices do not always align with SSC principles, instead replicating traditional donorship [1]. While China’s rhetoric on SSC has created large public expectations in African countries, the impact of the country’s efforts on sustainable development remains uncertain [35, 36]. To mitigate the potential negative impact of trilateral SSC disguised by the “value added” of trilateral cooperation and South‒South rhetoric, entrusting multilateral organizations to act as “neutral mediators and representatives for the recipient countries’ interests” has been proposed [18]. Accordingly, this case study on DRC–UNICEF–China project reveals empirical evidence of China’s potential DAH transformation facilitated by a multilateral organization, UNICEF.

## Methods

We conducted a qualitative case study of a DRC–UNICEF–China MNCH project. This project was implemented in the Miabi Health Zone in Kasai Oriental province in the DRC from 2020 to 2021 via an endowment from China’s South‒South Cooperation Assistance Fund (SSCAF, currently Global Development and South–South Cooperation Fund). We analyze data from project documents and seventeen semi-structured interviews using a pragmatic analytical framework based on the DAH program logic model and OECD’s trilateral cooperation framework.

### Case selection and study setting

We selected the DRC–UNICEF–China project (Table 1) as the case primarily because of the accessibility of new evidence via the independent study team’s (constituted by the authors) data collection and analysis, as well as knowledge generation and dissemination. The project is unique among the eight subprojects in the SSCAF-supported UNICEF project, titled “Improving Maternal, Newborn and Child Health in Eight African Countries”, as it is the only one to have conducted an independent evaluation post-implementation. Yet, as of April 2023, this project has not been listed in the OECD triangular cooperation repository of projects [37]. This study, therefore, constitutes the tip of the iceberg that the OECD, an advocate for trilateral cooperation, has not yet explored.

**Table 1:** Basic information on the DRC–UNICEF–China MNCH project

Project title		China–Africa collaboration to accelerate maternal, newborn, and child health in the Democratic Republic of Congo
**Geographical and population coverage**		170,000 residents, including 6800 pregnant women, 6800 newborns, and 34,000 children under 5 years in 14 health areas in the Miabi Health Zone in Kasai Oriental province
**Objectives**		(1) Develop capacity in the DRC for achieving Sustainable Development Goals (SDG) and the already-specified national or local MNCH policy goals. (2) Support Chinese partner agencies in developing their capacity to deliver health aid programs in selected African countries where the Chinese counterparts have a comparative advantage. (3) Support the development of long-term South‒South exchange mechanisms between China and selected African countries.
**Project activities**		(1) Capacity building for provincial managers for MNCH management (2) Capacity building for health workers at decentralized levels through mainstreaming and scaling clinical mentorship (3) Essential drug supply for MNCH at the community level (4) Rehabilitation of health facilities, including maternity wards (5) Promotion of Key Family Practices, including family planning
**Envisioned outcomes**		(1) Safer and cleaner delivery environment (2) Availability of essential drugs (3) Improved capacity among MNCH service providers (4) Improved community capacities for monitoring and mobilization (5) Improved coordination and management of health services (6) Lessons learned and good practices for potential scaling
**Duration**		January 1st, 2020, to December 30th, 2021, with a one-year no-cost extension.
**Funding**	Source	SSCAF of China
	Applicants	UNICEF
	Amount	US$ 1,000,000

Source: The project proposal and closure report, with authors’ amendments.

Abbreviations: ***DRC*** Democratic Republic of Congo; ***MNCH*** maternal, newborn and child health; ***SSCAF*** South‒South Cooperation Assistance Fund; ***UNICEF*** United Nations Children’s Fund.

A further aim of this study is to understand whether and how a trilateral cooperation modality for DAH benefits health and well-being. Our case was tasked with an important but challenging global health issue: MNCH. The DRC has faced a series of challenges to MNCH, including protracted armed conflicts, an underdeveloped public health system, and disrupted MNCH services. In 2016, large-scale armed conflict erupted in the Kasai region, where the DRC–UNICEF–China project was later implemented. This region experienced the most significant decline in antenatal care and skilled birth attendance coverage in the DRC, according to two Multiple Indicators Cluster Surveys in 2010 and 2017–2018 [38]. As MNCH is fundamental to fully realizing basic human rights, these humanitarian challenges have demanded a wider range of engagement from emerging partners to steer DAH toward greater equity, effectiveness, and sustainability. In the past four decades, China has made tremendous improvements in reducing maternal and child mortality. The global community has supported and recognized the country’s successes as a top performer in achieving Millennium Development Goals 4 and 5 [39]. In this sense, China has emerged as a Global South partner that can provide financing and, more importantly, the technical know-how to improve MNCH in other developing countries.

The DRC–UNICEF–China project also constituted a critical, progressive stage in China’s transition from DAH recipient to donor. Trilateral cooperation, as a new form of collaboration between China and UNICEF [40], has been adopted to utilize UNICEF’s global networks to pilot some of China’s best MNCH practices for MNCH development in the DRC. This project thus offers insights into how China has committed to its evolving role in global health, which may help inform other emerging development partners similarly engaged in trilateral SSC for health.

### Analytical framework

**Fig. 1:**
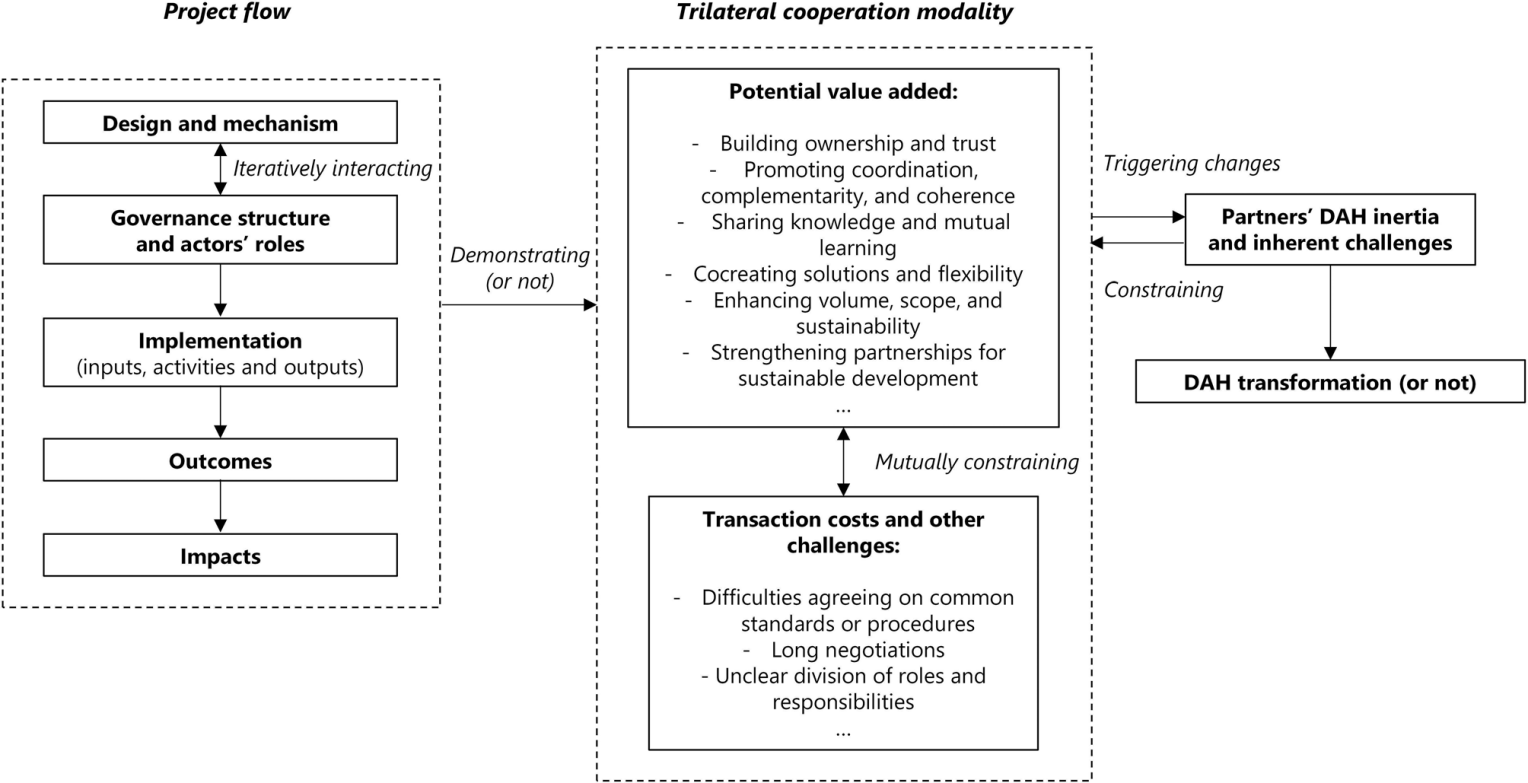
Analytical framework of the study. Abbreviation: ***DAH*** Development Assistance for Health

We developed a pragmatic analytical framework (Fig. 1) for the case study. This framework incorporated the logic model broadly used for DAH program management systems [41, 42]. We added two components iteratively interacting with each other in the project flow: “design and mechanism” [41] and “governance structure and actors’ roles”. This project flow helped identify how and why the project led to behavioral changes and corresponding health outcomes, potentially facilitating concrete analysis of how the trilateral cooperation modality functions throughout the project.

We then adopted OECD’s trilateral cooperation framework to understand the roles and responsibilities of each development partner. Under this framework, conventional trilateral cooperation involves (1) a “beneficiary partner”, who needs support to address obstacles to sustainable development, (2) a “pivotal partner”, who often has proven experience, knowledge, and expertise on the focal issue and shares these with others, and (3) a “facilitating partner”, who helps connect all partners and facilitates collaboration through existing transnational networks [7, 43]. Following this framework, previous studies have summarized deductive assumptions on trilateral cooperation’s value added and challenges [3, 5, 6]. We postulated that the project flow would demonstrate the value added in trilateral cooperation, which may trigger changes that counteract the partner’s (China, in this study) DAH inertia and address inherent DAH challenges [24, 28, 30, 44], thereby resulting in DAH transformation. However, given the multiple challenges that may constrain the transformative role of trilateral cooperation, another scenario where the trilateral cooperation modality does not transform DAH may also exist.

### Data collection

The data sources for this study consisted of project documents (obtained through personal correspondence and online queries; see Additional File 2) and interviews. Specifically, using purposive and snowball sampling methods, we recruited 21 respondents for seventeen interviews, including four interviews that had two respondents. The interviewees invited to this study represented the major project stakeholders. Table 2 lists the perspectives and roles of the respondents and the anonymization method. Respondents from the perspectives of the DRC and UNICEF DRC were Congolese, while others were Chinese.

**Table 2:** Perspectives and roles of the respondents and anonymization method

Perspective	Role	Respondents number	Anonymization
UNICEF	Officer at UNICEF DRC	1	UNICEF R3
	Specialist at UNICEF DRC	1	UNICEF R3
	Manager of the UNICEF DRC office in the project province	2	UNICEF R1, R2
	Officer at UNICEF China	2	UNICEF R4
DRC	DRC government	2	DRC government R1, R2
	Registered nurse (at health centers)	4	Registered nurse R1, R2, R3, R4
	Community health worker	5	Community health worker R1, R2, R3, R4
China	Government of China	2	China R1
	Experts	2	China R2, R3

Abbreviations: ***DRC*** Democratic Republic of Congo; ***UNICEF*** United Nations Children’s Fund

All the interviews were semi-structured and covered two groups of questions based on the analytical framework: (1) the project flow (e.g., “what has been delivered by the project” and “what have these efforts achieved?”); (2) the trilateral cooperation modality and partnerships in this project (e.g., “how did the trilateral partnership work” and “how did this partnership influence aid delivery?”). The authors tailored the interview guide for each respondent, considering their perspective and role.

All interviews took place between June 2021 to June 2022, typically lasting 30 to 90 min. Thirteen interviews were in French and four in Chinese; five were conducted online due to COVID-19 travel restrictions, and others were face-to-face. All interviews were recorded, transcribed, and carefully reviewed; the French transcripts were translated into English for analysis.

### Data analysis

The authors uploaded the interview transcripts and project documents to the qualitative analysis software MAXQDA 2022 (Release 22.3.0) and analyzed all the data using a framework approach [45, 46]. The actual process involved five steps: (1) data familiarization; (2) development of a coding scheme through pilot coding for randomly selected transcripts and documents using the analytical framework (Fig. 1); (3) independent data coding using the coding scheme by two of the authors, with additional codes identified and defined when necessary, and inductive identification of recurrent and important themes and subthemes surrounding trilateral cooperation; (4) data summary and comparison; and (5) data synthesis and evaluation of the relationships and interactions among the themes and subthemes. This paper presents these results according to the defined themes.

## Results

### Exploiting comparative advantages to address development needs

**Fig. 2:**
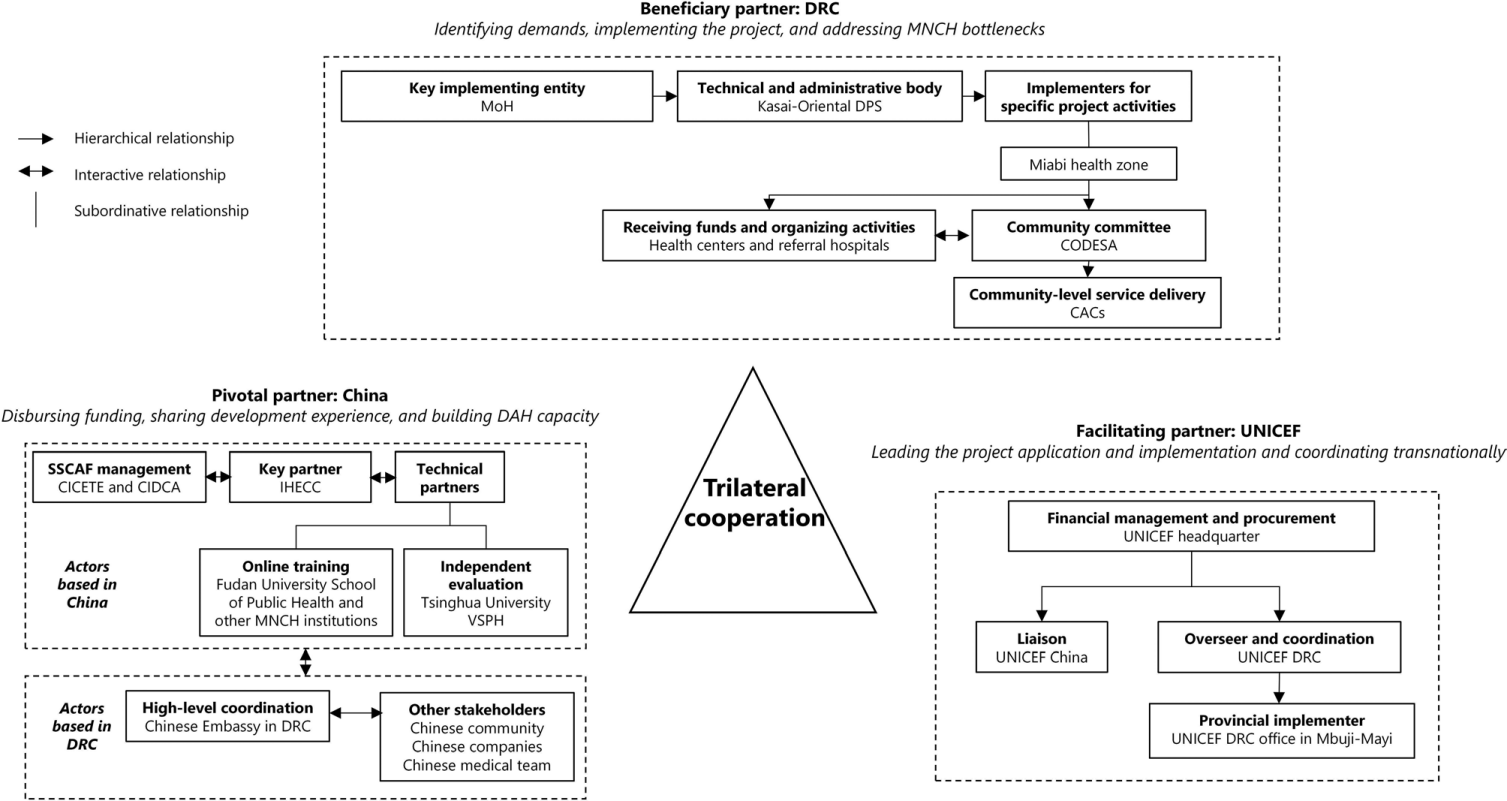
Governance structure and actors involved in the DRC–UNICEF–China MNCH project Abbreviations: ***CICETE*** China International Center for Economic and Technological Exchange; ***CAC*** Community Action Group; ***CIDCA*** China International Development Cooperation Agency; ***CODESA*** Health Area Development Committee; ***DAH*** development assistance for health; ***DPS*** Provincial Health Division; ***DRC*** Democratic Republic of Congo; ***IHECC*** International Health Exchange and Cooperation Center; ***MoH*** Ministry of Health; ***MNCH*** maternal, newborn and child health; ***SSCAF*** South‒South Cooperation Assistance Fund; ***UNICEF*** United Nations Children’s Fund; ***VSPH*** Vanke School of Public Health.

The complex, multilayered project governance structure embodied the comparative advantages of each partner. We summarized this structure (shown in Fig. 2) based on project documents and interviews from all perspectives.

The “beneficiary partner”—the DRC—primarily designed and implemented the project. The Ministry of Health (MoH) was the key implementing entity responsible for project initiation, negotiation, and oversight. The Provincial Health Division (*Division Provincial de la Santé;* DPS) was the technical and administrative body for project implementation. Specific project activities involved the health zone, health centers and referral hospitals, Health Area Development Committee (*Comité de Développement de l’Aire de Santé*; CODESA), and community action groups (*Cellules d’Animation Communautaire*; CAC).

The “pivotal partner”—China—disbursed SSCAF funding and provided technical assistance while improving its institutional capacity for DAH delivery. The International Health Exchange and Cooperation Center (IHECC) of the National Health Commission—the leading implementer—provided technical support and coordinated with other Chinese technical partners. The IHECC commissioned Fudan University’s School of Public Health and other MNCH institutions to provide online training courses. Tsinghua University’s Vanke School of Public Health (VSPH) acted as another technical partner, conducting an independent evaluation. The China International Center for Economic and Technological Exchange (CICETE) was the SSCAF management office, operating under the Chinese Ministry of Commerce. CICETE’s SSCAF management responsibilities were gradually passed over to the China International Development Cooperation Agency (CIDCA), established in 2018. In the DRC, the main Chinese stakeholders were the Chinese Embassy, the Chinese companies based in Miabi, and the local Chinese community.

The “facilitating partner”—UNICEF—led the application and implementation of the project “in close partnership with” the other two partners. Endowed with an on-site office and MNCH expertise, it acted as a coordinator linking all stakeholders at the trilateral, national, provincial, health zone, and community levels for information exchange and solution co-creation. UNICEF headquarters functioned as the highest “manager” on the UNICEF side, especially for financial management and procurement; UNICEF China liaised with Chinese counterparts to ensure proper information exchange on the project’s progress; and UNICEF DRC (including the Kinshasa office and the office in Mbuji-Mayi, the capital of the Kasai Oriental province) oversaw the project and coordinated with DRC implementing partners.

#### Amplifying the beneficiary’s ownership

The abovementioned complementarity enabled the project to amplify the beneficiary’s ownership and autonomy, thereby creating opportunities to address the consistent lack of beneficiary ownership during and post-DAH, well-documented in China’s traditional, bilateral DAH [30]. The DRC government displayed great accountability, initiating project planning and bearing responsibility for implementation. Previously, the government had displayed “lethargy” (*UNICEF R2*) in some UNICEF projects supported by other donors in the DRC. In contrast, the closure report found a “strong commitment shown by the DRC Ministry of Health” to this project. According to respondents at the provincial and operational levels, DPS and health zone executives carried out project activities. They worked to align these activities with DPS efforts, which helped pave the way for the continuation of project-supported activities post-project. At the community level, due to UNICEF’s longstanding presence in local community empowerment programming, the trilateral governance structure also strengthened community ownership on a multisectoral level. Specifically, most actors at the community level, including village chiefs, community health workers, as well as heads and administrators at the health zone office and health centers, participated in the management committee and addressed cross-sectoral issues. This multisectoral approach to mobilizing multiple stakeholders was deemed a “big step forward” in the local health system (*Registered Nurse R3*).

#### Aligning with the local health system

The project was designed as part of the DRC’s national health development strategy and integrated into the local health system—a departure from past Chinese DAH projects that were not sufficiently demand-driven in design [24, 30, 44]. The project proposal noted that, following the initiation of the project design, the DRC MoH and UNICEF recognized the importance of strengthening the health system at the community level. Due to the prolonged armed conflict within the region, the decentralized health system in Miabi has heavily relied upon community health workers (*relais communautaires;* RECOs) to deliver daily MNCH services [47]. Thus, in addition to the essential drugs, equipment, and facility rehabilitation that China typically provides in its bilateral DAH [27], the project also focused on capacity building for MNCH service providers. From provincial, zonal, and facility-level health managers to RECOs, the project provided essential technical tools developed by Chinese MNCH institutes. As such, the DRC and UNICEF respondents asserted that the project helped strengthen health workers’ capacities for data collection and analysis, routine monitoring of MNCH activities, community mobilization, and MNCH awareness building.

#### Synergizing with existing donors’ projects

The project engaged China for aid coordination among development partners and helped prevent aid fragmentation [21] to a certain extent. UNICEF and DRC respondents reported that a coordination mechanism was used to harmonize projects supported by external partners, including UNICEF, the European Union (EU), the United States Agency for International Development, etc. The DRC–UNICEF–China project complemented preexisting EU funding to support the overall functioning of the local health system. Monitoring activities were also based on the framework of the UNICEF-initiated Child-Friendly Community project, launched in 2017. While these two projects were distinct at the management level, “everything was integrated at the community level” (*UNICEF R2*). The Child-Friendly Community project helped establish dynamics between UNICEF and the community, which provided a foundation for community engagement that was revitalized during the implementation of the DRC–UNICEF–China project:

*“The [DRC–UNICEF–China] project targeted an area which already, of course, with UNICEF, made progress in the context of community dynamics; we had already integrated the approach of community life in the health zone, and the project cemented or practically revitalized this approach further in the health zone.” (DRC government R1)*

### Harmonizing rules and procedures

**Fig. 3:**
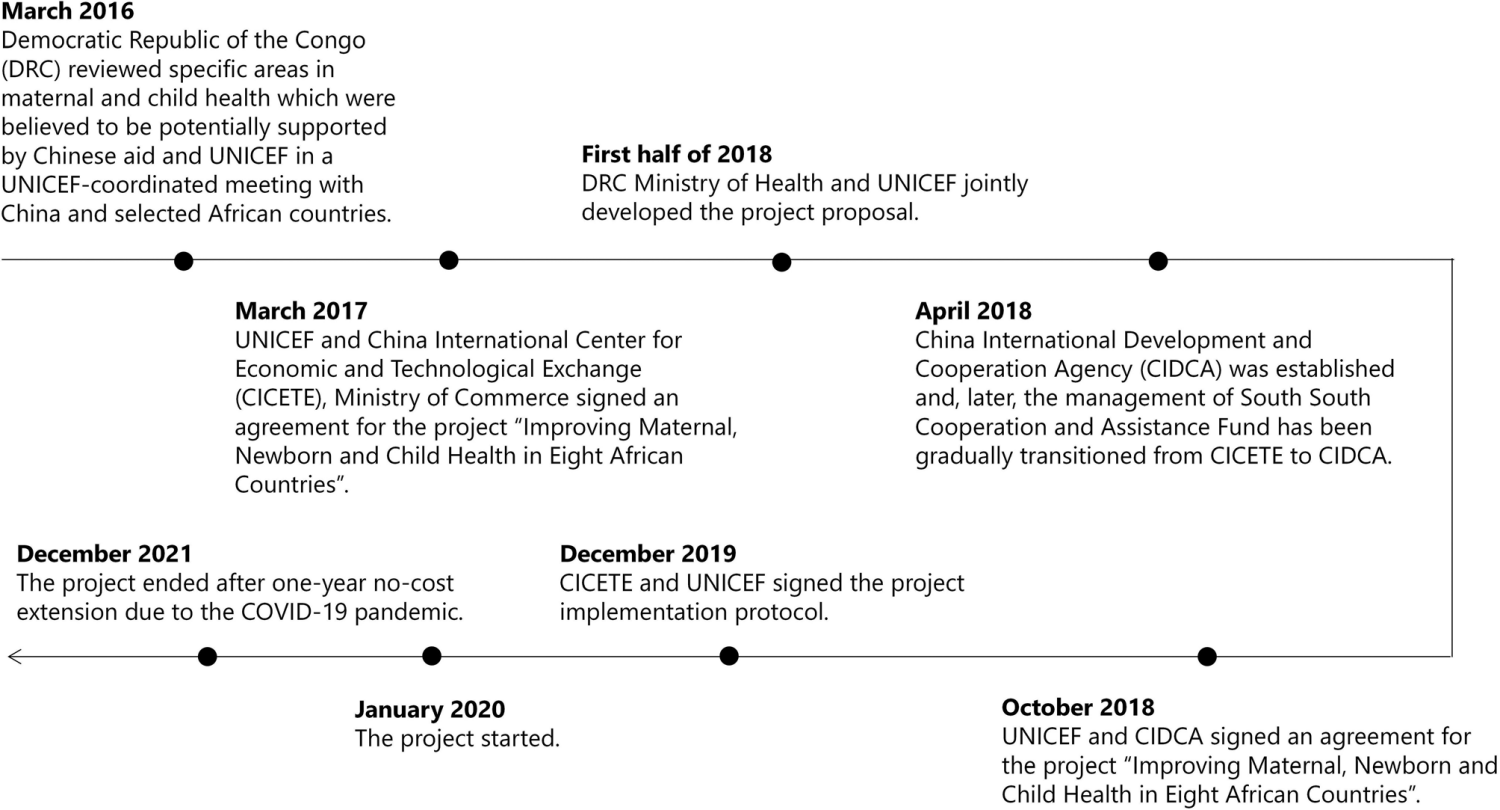
Timeline of the DRC–UNICEF–China MNCH project. (Abbreviations: ***CICETE*** China International Center for Economic and Technological Exchange; ***CIDCA*** China International Development Cooperation Agency; ***DRC*** Democratic Republic of Congo; ***SSCAF*** South‒South Cooperation Assistance Fund; ***UNICEF*** United Nations Children’s Fund)

The negotiation underwent a lengthy design stage. We summarized the project timeline in Fig. 3, according to respondents, the project proposal, and the closure report. A UNICEF respondent noted that the project entered a new space without any preexisting, workable governance infrastructure among the three partners. Given that each side had its own practices and procedures, China and UNICEF had to bridge discrepancies between the different project management standards and norms. Thus, it took a long time to determine and clarify the roles and responsibilities of the respective partners:

*“China has accumulated decades of experience in bilateral aid and has its own set of mature practices. So, how can the mature rules and regulations of China’s bilateral aid be aligned with the rules and regulations of the UN agencies? Even if the UN agencies have a common institutional framework, each UN agency is different. So, there were collisions, and it was a very difficult process to align… While the Chinese government had a proactive, cooperative attitude towards learning the rules and regulations of UNICEF, UNDP, and UNFPA, the process was longer [than we expected].” (UNICEF R4)*

The negotiation process, while time-consuming, helped each partner better understand and learn from one another. After rounds of arduous negotiation, China agreed to generally follow the UN’s rules and regulations, according to Chinese and UNICEF respondents. A UNICEF respondent and the closure report also noted that China insisted on quarterly reviews, which were more frequent than the standard UNICEF proposed, to emphasize project result documentation and robust monitoring. The project proposal ultimately adopted UNICEF rules and regulations for procurement and cash transfers, as well as a quarterly review schedule, signaling a harmonization of rules and regulations between Chinese and international standards. Through this harmonization, Chinese and UNICEF respondents indicated that key institutions and personnel in Chinese DAH gained more familiarity with the rules and procedures of UNICEF and the UN system. Likewise, UNICEF also better understood China’s project management standards.

### Institutionalizing mutual learning and knowledge sharing

As part of the “SSCAF Training for Maternal, Newborn and Child Health Project” jointly executed by the IHECC and UNICEF in eight African countries, the training in the DRC–UNICEF–China project fostered a new model for technical assistance in China’s DAH, one that encourages “demand-oriented training” (*China R1)* and “mutual learning” (*China R2 and R3*). The participants, from the DRC and seven other African countries, were health managers and practitioners recommended by UNICEF. Through a needs assessment conducted among the participants, the training facilitated a comprehensive match-making process of the demand and supply of MNCH development knowledge. Furthermore, continuous efforts to encourage interaction and feedback led to a second round of training courses in response to newly emerged demands, which helped build sustained partnerships for mutual learning and knowledge sharing among Chinese and African health professionals. Chinese respondents believed such interactive experiences and often interchangeable teaching–learning relationships have helped to generate ideational change among Chinese experts.

Chinese respondents also reported that the training strongly encouraged knowledge generation based on China’s development progress and MNCH experience in recent decades. With UNICEF’s technical assistance, Chinese technical partners systematically investigated the country’s MNCH achievements and identified the best practices at various development stages. This summative process of knowledge generation followed the WHO framework of the “six building blocks” of the health system, which rendered it both familiar to global audiences and empirically situated in China’s MNCH development. The training process demonstrated that “sharing development experience” was conceptualized as the epitome of the spirit of SSC. In this training, China’s MNCH experience sharing entailed not a linear process of condensing and replicating a universal development model, but rather a systematic approach seeking to fully understand the sociocultural preconditions of development and engage in a dynamic and analytical problem-solving mindset to address underdevelopment.

Supported by UNICEF and IHECC, the training institutionalized workable forms of multi-stakeholder cooperation. Chinese respondents observed that it mobilized a wide array of Chinese MNCH institutions and experts to address the training needs of the eight African countries, thereby strengthening talent cultivation and multi-stakeholder cooperation in the Chinese DAH system. This training also created valuable opportunities to equip Chinese MNCH professionals, especially young talents from universities, research institutes, maternity and child health care hospitals, community health centers, overseas CMTs, etc., with a comprehensive skill-set comprising of language, teaching, organizing, etc. Chinese respondents revealed the opportunity for institutionalizing China’s future technical MNCH assistance programs:

*“Through this training, we are now confident and hopeful that this modality of multi-stakeholder cooperation in trilateral partnership will continue. While continuously engaging with UNICEF, we also want to use resources from the Chinese Ministry of Commerce and other national DAH funds to sustain our MNCH training programs in China and abroad…There are also plans to have our overseas medical teams with obstetrics and gynecology equipment establish training posts in African countries. We always emphasize demand-driven training and mobilize all stakeholders as well as capacity building among our health professionals to sustain these training programs.” (China R1)*

### Increasing China’s visibility as an emerging DAH partner

UNICEF DRC respondents indicated that, prior to the project, the local people had received little first-hand information about China besides that a Chinese mining company was located there. According to the local respondents, China’s visibility as a development partner increased among community workers and the local population (especially women) through project implementation. A niche for China’s DAH then emerged: given that the local community may have grown “tired of” traditional donors (*Registered Nurse R4*) and that China has undergone a similar trajectory in MNCH development as the DRC over the past few decades, a more proactive transfer of China’s development experience to the DRC may be beneficial:

*“You should know that the Chinese are not only exporting diamonds; they also have technical skills that they can transfer to the DRC. China was like the DRC today. And, even on the medical level, [you should know] how they were at one time and took charge of maternal health in their country. Why not transfer these skills and the Chinese model to develop our health system?” (UNICEF R2)*

UNICEF was the key actor advocating this niche. As the quarterly and closure reports documented, UNICEF published social media posts on its Facebook and Twitter pages and sent promotional materials to the field during project implementation. According to the project reports, it also held several meetings with Chinese stakeholders, including the Embassy of China, CMT, Chinese WHO health experts, a Chinese mining company, and other companies. Therefore, utilizing UNICEF’s communication platforms to disseminate project-related information helped increase the visibility of China’s DAH projects, both in the DRC and China as well as globally.

### Neglecting key stakeholders in the complex governance structure

While the trilateral governance structure helped amplify the beneficiary’s ownership, DPS and UNICEF respondents revealed that the *de facto* involvement of DPS in project decision-making was weak. Normally, DPS manages almost all projects in the province due to the country’s large geographic size and decentralized health system. However, the project’s decision-making and main project documents were held in Kinshasa, the DRC’s capital. Provincial leaders were not involved in project design and planning, which led to misalignment between certain project interventions and the local conditions during implementation. As a result, project outcomes were partially weakened, for example, for rehabilitation:

*“We were only told—we came to rehabilitate, we came to build—and without ever associating ourselves from the beginning…We wished that a building in the hospital courtyard had been thoroughly rehabilitated so that it could be used for gynecology and obstetrics… This rehabilitation was not even completed in all the buildings; it was just the delivery room... We say to ourselves that this is a project where we have not been integrated… So, at least, we have to stay together; even if things are dealt with at the national level, [they should] also listen to those on the ground and consider [these suggestions] to improve things.” (DRC government R2)*

### Pivotal partner’s local absence

While UNICEF’s local networks in the DRC facilitated the engagement of community health actors for the project, the responsibility of supporting local DRC partners was solely entrusted to UNICEF; China was deemed merely a “donor” (*DRC government R1*) with little in-country involvement. Chinese counterparts’ presence and activities, such as routine project management and periodic supervision, failed to penetrate the project site in the Miabi Health Zone deeply. According to the quarterly reports, the three high-profile meetings in Kinshasa occurred only at the beginning of project implementation (the first quarter of 2020). In addition, although the MNCH training led by the IHECC offered a promising gesture for mutual learning from China, it was conducted online without on-site activities due to the COVID-19 pandemic.

*“The Chinese side stayed much longer in Kinshasa with the UNICEF ​​Kinshasa office and perhaps [paid] a visit to the Ministry of Health. At the community level, many people don’t even know that the Chinese government has funded a good amount of $ 1 million for health development in the area… The Chinese government, through its Schools of Public Health, could be involved down to the grassroots level in the province.” (UNICEF R2)*

Thus, while the ideals of South‒South and trilateral cooperation call for equal standing amongst all partners, China, due to its local absence, nevertheless remained a remote “donor” in this project. This remoteness may have prevented China from gaining real-time knowledge of the local contexts of beneficiaries and the evolving needs for sustainable, effective coverage—a common issue documented in China’s previous DAH efforts [24, 30].

### Demanding high transaction costs while ensuring transparency

**Fig. 4:**
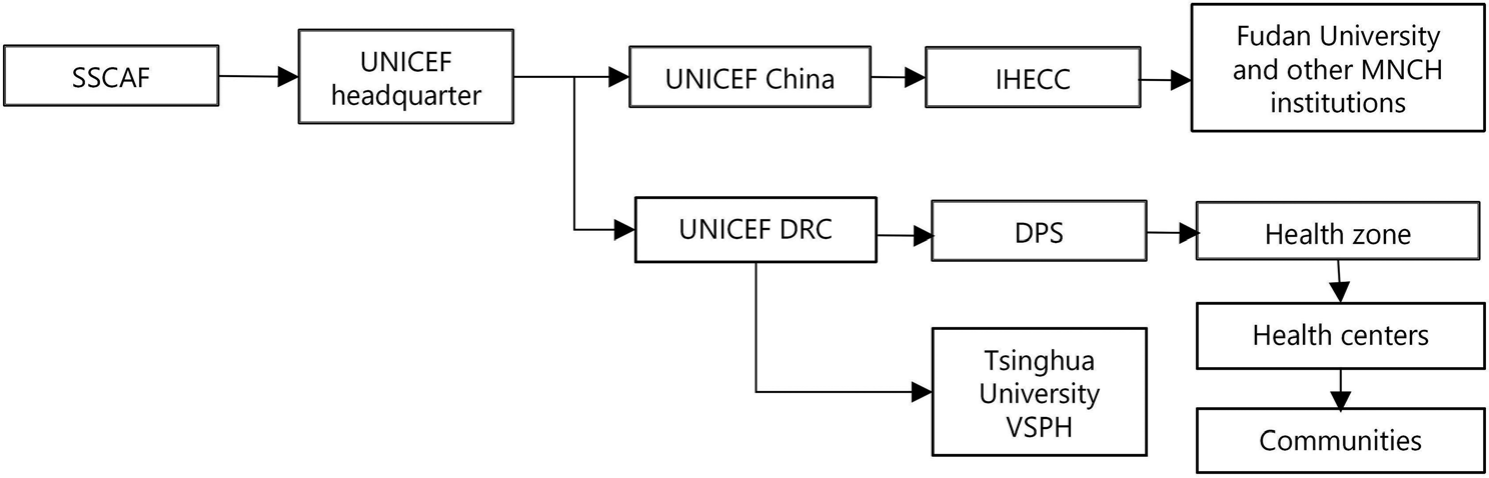
Financial flow in the DRC–UNICEF–China MNCH project. (Abbreviations: ***DPS*** Provincial Health Division; ***DRC*** Democratic Republic of Congo; ***IHECC*** International Health Exchange and Cooperation Center; ***MNCH*** maternal, newborn and child health; ***SSCAF*** South‒South Cooperation Assistance Fund; ***UNICEF*** United Nations Children’s Fund; ***VSPH*** Vanke School of Public Health)

We summarized the financial flow (Fig. 4) based on project documents and interviews with UNICEF respondents. The flow illuminates how the trilateral governance structure enabled transparency while incurring high transaction costs. In contrast to projects where donors execute funds directly, UNICEF headquarters received the SSCAF funds and then allocated them to UNICEF China and the DRC. According to the project proposal, UNICEF China oversaw the channeling of funds for activities undertaken by the IHECC. In the DRC, DPS sent funding requests and received funds from UNICEF DRC, both for quarterly DPS monitoring and for transmission to the health zone. The health zone then carried out zonal activities while sending funds to health centers for organizing project activities at the community level. UNICEF DRC, acting as a safeguard and “external watcher” (*UNICEF R1*) to prevent “problems with security and transparency on the government side” (*UNICEF R2*), made programmatic visits to monitor the financial management at each operational level, sending relevant reports to the Chinese counterparts. While ensuring “security and transparency” (*UNICEF R2*), this financial arrangement incurred high administrative costs and was time-consuming, as observed in this short, two-year project. Similarly, respondents also revealed that the procurement arrangements within the UNICEF bureaucratic system delayed project implementation.

## Discussion

### Opportunities

A trilateral SSC governance structure that exploits comparative advantages [6, 21, 48] can cultivate a demand-oriented approach for aid delivery and facilitate a better alignment of resource provision with health development needs [49]. In the case of the DRC–UNICEF–China project, strengthening the beneficiary’s ownership for post-DAH sustainability, aligning with the local health system, and synergizing with existing donors’ projects helped counter China’s DAH inertia by addressing the neglect of beneficiary partner’s ownership, limited identification of the beneficiary’s needs and goals, and infrequent collaboration with other donors, respectively [24, 30, 44, 50].

Looking beyond common concerns about the transaction costs of a single trilateral cooperation project [5, 6, 43], our study indicates that early trilateral projects may pave the way for reducing the transaction costs (e.g., long negotiations) of future projects over time, by harmonizing rules and procedures and institutionalizing mutual learning and knowledge sharing. For emerging development partners like China that actively seek to establish effective partnerships for DAH projects, the precedents set by early projects for dialogue and harmonization of rules and procedures may help guide future DAH projects involving global health regimes—opportunities far beyond what traditional South–South DAH approach can offer. Trilateral partnerships can also set the stage for emerging development partners to translate their highly efficient domestic health practices into global evidence and best practices and to professionalize their technical assistance. In the DRC–UNICEF–China project, Chinese respondents indicated growing consensus on the importance of knowledge sharing and mutual learning among Chinese DAH managers and practitioners involved, who are also well-positioned to share the knowledge acquired from UNICEF with a global audience. These opportunities can facilitate institutional and human resources technical capacity building for pivotal partners, which, in turn, can promote DAH transformation [5, 43]. In China’s case, trilateral cooperation can also help address documented challenges to its DAH, such as the lack of institutional support for the beneficiary countries’ individual and professional needs [27, 30, 31, 51].

Additionally, effective public outreach through multilateral organizations can help spread awareness to local beneficiaries and the global community of development professionals on how emerging development partners can contribute to global health through the transfer of development experience and knowledge [1, 5]. Concerning China’s strategic interests, while the DRC–UNICEF–China project indeed provided an opportunity to transform the image of China’s DAH in global discourse [52], increased visibility can also raise confidence among Chinese DAH policy-makers of the value of deepening global health engagements through China–Africa SSC and partnerships with multilateral organizations. More broadly, trilateral cooperation may provide opportunities for other emerging countries to gain global recognition as potential partners that can provide alternatives for health development cooperation while avoiding the pitfalls of traditional DAH [31].

### Challenges

The DRC–UNICEF–China project highlights a fundamental challenge in trilateral cooperation: how complex governance structures can hinder sufficient involvement of some key stakeholders [6, 51], which in turn impairs beneficiary ownership [14]. In particular, where facilitating partners take on leading roles, power asymmetries can emerge, damaging the principle of ownership and reproducing the same shortcomings as traditional North–South relationships [1]. While limited communication between the MoH and DPS may have been a preexisting contextual issue, the risk this weak link between these two actors posed to the project governance structure was neglected. Therefore, despite its long presence in the DRC, UNICEF could not ensure full context-based project design and implementation. Taken together, this case calls attention to how the entrustment of facilitating partners can inadvertently perpetuate uneven power relations if care is not taken to curtail such hierarchies [14].

While an emerging development partner may utilize the local networks of multilateral organizations or other partners in trilateral cooperation projects to substitute the absence of on-site offices [5, 50, 52], such arrangements may challenge the emerging development partner’s long-term DAH engagement. Indeed, being removed from the subtle but critical local issues can limit pivotal partners’ participation in project decision-making [51, 53] and reduce their involvement to a one-off programmatic activity, as opposed to “accompanying the intended beneficiaries” [43] for sustainable impacts on local health systems. Thus, fully realizing the transformative opportunities of trilateral cooperation requires emerging development partners to firmly commit to deepening on-site participation in the beneficiary countries.

In addition, the case revealed how a lengthy project management flow, while ensuring transparency, nevertheless poses a challenge to effective resource distribution by incurring high transaction costs, which in turn can limit the resources available for programmatic activities and compromise overall project effectiveness—to a greater degree than is generally encountered in bilateral, direct projects.

### Implications

This study echoes some of the findings in trilateral SSC literature that claim power structures and philanthropic, normative justification for health equity are often juxtaposed in trilateral SSC partnerships [1, 14, 54]. It found that opportunities for transformation brought forth by the DRC–UNICEF–China project can be understood as part of China’s long cognitive learning process toward international engagement [21, 22], rooted in China’s deeper ambition to promote itself as a “responsible” development partner globally [10, 11, 21, 22]. The challenges identified here imply the need to be wary of how the essentialized identity of trilateral SSC, or assuming “a neutralized alliance for mutual understanding”, may obscure power hierarchies [1, 14].

Therefore, strategic contestation, negotiation, and accommodation by stakeholders [1, 55, 56] for achieving shared responsibility [14, 57] should be seen as fundamental to trilateral SSC. To embrace the transformative role of trilateral cooperation, partners should avoid viewing trilateral SSC as merely “a temporary way to obtain specific development outcomes” [1]. Rather, trilateral SSC should be understood as a process of building durable development partnerships beyond the specific disease or health area (in this case, MNCH), and even beyond the health sector altogether. With such conditions in place, China and other emerging development partners—as pivotal partners in global health trilateral SSC—may have strong potential to play intersectional and mutually reinforcing roles in accelerating the SDGs. They can act as middle-income countries providing new sources of development financing, top-performing partners transferring development experiences, and, finally, Global South advocates seeking more inclusive, equal, and effective long-term DAH models.

This study thus suggests that efforts from all development partners are needed to further transform trilateral cooperation and DAH. Such efforts must include (1) strengthening the beneficiary partner’s ownership at all levels, especially in the context of decentralized health systems; (2) engaging the emerging pivotal partner to better understand the beneficiary partner’s local context(s) and needs; and (3) ensuring that the resources and partnerships to support programmatic activities and the health and well-being of the beneficiaries are available, even in short-term pilot projects.

### Limitations

This study has some limitations. We were unable to recruit respondents from the DRC MoH, UNICEF headquarters, and MNCH service users in Miabi Health Zone who had received project support. Nonetheless, the existing list of interviewees covers the majority of relevant stakeholders and largely reflects both the big picture and details of the project. Moreover, the CIDCA was only established in 2018, and the agency’s responsibilities and roles remain unclear. Therefore, this study failed to clarify its role in the project. In addition, as the project only ended in 2021, the long-term impacts remain challenging to identify.

## Conclusion

Evidence from the DRC–UNICEF–China MNCH project unfolds a complicated, multifaceted story in the theme of “the emerging development partner’s DAH transformation facilitated by a multilateral organization”. This study suggests that trilateral SSC for health can provide transformative opportunities for emerging development partners’ DAH to generate and deliver context-based, demand-oriented solutions, harmonize rules and procedures, institutionalize mutual learning and knowledge sharing, and increase the visibility of emerging development partners as sources for South‒South development experience transfer. This study also warns of the potential challenges arising from the complex governance structure and entrustment of facilitating partners in trilateral projects. We thus call for strengthening the beneficiary partner’s ownership at all levels, engaging the emerging pivotal partner to better understand the beneficiary partner’s local context(s) and needs, and ensuring available resources to support programmatic activities and long-term partnerships for the health and well-being of the beneficiaries. Deepening understanding of trilateral cooperation and DAH transformation involving China and other Global South countries requires more public data and broader scholarly engagement to support more concrete, sophisticated empirical studies.

## Electronic supplementary material

Below is the link to the electronic supplementary material.

Supplementary Material 1

## Data Availability

Upon the request of IRB, interview transcripts used for the analysis are confidential. Additional file 2 lists project documents as data sources of this study; the documents obtained via personal correspondence should be addressed to UNICEF and IHECC. The detailed results of the review of related literature (see search strategy and the short review in Additional file 1) are available from the authors, upon reasonable request.

## Abbreviations

CICETE: China International Center for Economic and Technological Exchange
CIDCA: China International Development Cooperation Agency
CAC: *Cellules d’Animation Communautaire*, Community Action Group
CODESA: *Comité de Développement de l’Aire de Santé*, Health Area Development Committee
CMT: Chinese medical team
DAH: development assistance for health
DPS: *Division Provincial de la Santé*, Provincial Health Division
DRC: Democratic Republic of Congo
EU: European Union
IHECC: International Health Exchange and Cooperation Center
MoH: Ministry of Health
MNCH: maternal, newborn and child health
RECO: *relais communautaires*, community health workers
SDGs: Sustainable Development Goals
SSCAF: South‒South Cooperation Assistance Fund
UN: United Nations
UNICEF: United Nations Children’s Fund
VSPH: Vanke School of Public Health
WHO: World Health Organization
