# Supplementary material for: Opportunities and challenges of trilateral South‒South cooperation for transforming development assistance for health: evidence from a DRC–UNICEF–China maternal, newborn, and child health project
Source: Global Health. 2023 Jun 8;19:37. doi: 10.1186/s12992-023-00934-9 (PMC10249568; doi:10.1186/s12992-023-00934-9)
Supplement: Supplementary file 1 — Supplementary Material 1 [file 12992_2023_934_MOESM1_ESM.docx]

# Additional file 1. Review of literature on South-South and trilateral cooperation literature

Table S1. Search strategy, eligibility criteria and screening results

| Source of literature | Web of Science core collection |
| --- | --- |
| Search query | TS=((trilateral or triangular or tripartite) NEAR/5 (cooperat* or collaborat* or assistance or aid)) OR TI=((trilateral OR triangular OR tripartite) AND (cooperat* OR collaborat* OR assistance OR aid)) |
| Publication date | All years |
| Search date | 13 April 2023 |
| Query link | https://www.webofscience.com/wos/woscc/summary/6cefd4d7-c08a-4c92-9a7c-ac4c754849e3-8187c1cb/relevance/1 |
| Search results | 507 |
| Inclusion criteria | The main topic is trilateral cooperation encompassing South-South cooperation for development assistance |
| Exclusin criteria | 1. Trilateral cooperation is not the main topic 2. South-South cooperation is not touched upon 3. The purpose is not for development assistance, e.g., economic or security cooperation, humanitarian aid, environmental governance, scientific capacity building, etc. 4. The trilateral cooperation is not transnational or international cooperation among three parties, but rather one involving more than one domestic parties. 5. Without English or Chinese abstracts 6. Commentary, letter or editorial |
| Title and abstract screening results | 29, among which 8 were about China |

Descriptive mapping of trilateral cooperation in Africa [1] and Latin America [2] and China’s engagement has been performed [3,4], and there are case studies of trilateral projects involving Global North countries [5,6] and China [7,8]. These studies have provided real-world evidence—mapping a general picture or narrating specific cases—but they are rather descriptive or narrative. Some scholars have conducted conceptual and prospective research primarily analyzing the cooperation foundations and policy considerations of the trilateral cooperation involving Global North countries [9–13] and China [14–19]. These studies have laid the foundation for analyzing trilateral cooperation in a deductive manner. More relevant to this paper, studies on the transformative role of trilateral cooperation have discussed partnership transformation involving Southern countries [20,21]. Zhang’s research on China’s trilateral cooperation, for instance, presents an insightful analysis revealing China’s development assistance (including DAH) transformation [22,23], although the author’s focus is not on transformation. A study also summarized how the China–UK health partnership transformed from a bilateral to a trilateral approach, providing a valuable perspective for examining China’s DAH transformation in its early stage, when China had just embarked on graduating from DAH recipient to donor [24]. Building on these works, this study provides recent evidence through a case study of a project involving a multilateral organization, which has been rarely touched upon in the abovementioned literature, and concentrates on the transformative role of trilateral cooperation in DAH.

The search of this review of literature has been saved in searchRxiv (doi/10.1079/searchRxiv.2022.00066).

# References

[1] Nowak W. Triangular development cooperation in African countries, 2018.

[2] Burges S. South-South and triangular cooperation in Latin America: affirmative policies and practices transformed. Int Aff 2013;89:1363–4.

[3] Chao Z. China’s Triangular Development Cooperation: Perception and Practices. China Rep 2021;57:169–91. https://doi.org/10.1177/00094455211004046.

[4] Prantz S, Zhang X. Triangular Cooperation: Different Approaches, Same Modality. IDS Bull 2021;52. https://doi.org/10.19088/1968-2021.125.

[5] Schmink M, Cairns Smart J, Sitoe I, Bowen W, Silva HR, Ecole CC, et al. Challenges and opportunities of trilateral co-operation: Collaboration by the USA, Brazil and Mozambique on horticultural research, 2011–2015. Dev Policy Rev 2020;38:575–93. https://doi.org/10.1111/dpr.12437.

[6] Hosono A. Scaling Up South-South Cooperation through Triangular Cooperation: The Japanese Experience. 2013.

[7] Zhang D. China–Australia–Papua New Guinea Trilateral Aid Cooperation on Malaria Control. The Australian National University; 2016.

[8] Ma X, Lu S, Wang D, Zhou Z, Feng J, Yan H, et al. China-UK-Tanzania Pilot Project on Malaria Control. China CDC Wkly 2020;2:820–2. https://doi.org/10.46234/ccdcw2020.179.

[9] Parlar Dal E, Dipama S. Assessing the EU’s and Turkey’s Triangular Development Cooperation partnership in the Sub-Saharan Africa. Alternatives 2022;47:209–34. https://doi.org/10.1177/03043754221135411.

[10] Abdenur A, da Fonseca J. The North’s Growing Role in South-South Cooperation: keeping the foothold. THIRD WORLD Q 2013;34:1475–91. https://doi.org/10.1080/01436597.2013.831579.

[11] Lengfelder CS. Why Triangular Development Cooperation? Germany and the Emerging Powers. Ger Polit 2016;25:1–24. https://doi.org/10.1080/09644008.2015.1024239.

[12] Kim S. Rendering (in-)visible?: analysing the formation of Japan’s Triangular Development Cooperation in Southeast Asia. Globalizations 2022;19:1068–87. https://doi.org/10.1080/14747731.2021.2025300.

[13] Raposo P, Raposo P. Japan’s South-South Cooperation and Triangular Cooperation in Africa: Implications for TICAD. 2014. https://doi.org/10.1057/9781137493989.0010.

[14] Lei W. China and the United States in Africa Competition or Cooperation? CHINA Q Int Strateg Stud 2020;6:123–41. https://doi.org/10.1142/S2377740020500037.

[15] Wissenbach U. The EU’s Response to China’s Africa Safari: Can Triangular Co-operation Match Needs? Eur J Dev Res 2009;21:662–74. https://doi.org/10.1057/ejdr.2009.25.

[16] Wei S. Development Cooperation in Africa Creating New Momentum for China-U.S. Relations. CHINA Q Int Strateg Stud 2018;4:577–94. https://doi.org/10.1142/S2377740018500252.

[17] Hooijmaaijers B. China’s rise in Africa and the response of the EU: a theoretical analysis of the EU-China-Africa trilateral cooperation policy initiative. J Eur Integr 2018;40:443–60. https://doi.org/10.1080/07036337.2018.1465418.

[18] Zhang D. A tango by two superpowers: China-US cooperation in trilateral aid and implications for their bilateral relations. ASIAN J Polit Sci 2018;26:181–200. https://doi.org/10.1080/02185377.2018.1462218.

[19] Armel K. Trilateral Cooperation China’s and the EU’s Foreign Assistance in Africa. CHINA Q Int Strateg Stud 2020;6:311–32. https://doi.org/10.1142/S2377740020500189.

[20] McEwan C, Mawdsley E. Trilateral Development Cooperation: Power and Politics in Emerging Aid Relationships. Dev CHANGE 2012;43:1185–209. https://doi.org/10.1111/j.1467-7660.2012.01805.x.

[21] Alonso JA, Santander G. Triangular Cooperation: Change or Continuity? Eur J Dev Res 2022;34:248–71. https://doi.org/10.1057/s41287-021-00370-8.

[22] Zhang D. A Cautious New Approach: China’s Growing Trilateral Aid Cooperation. 1st ed. ANU Press; 2020. https://doi.org/10.22459/CNA.2020.

[23] Zhang D. Why cooperate with others? Demystifying China’s trilateral aid cooperation. Pac Rev 2017;30:750–68. https://doi.org/10.1080/09512748.2017.1296886.

[24] Wang X, Liu P, Xu T, Chen Y, Yu Y, Chen X, et al. China-UK partnership for global health: practices and implications of the Global Health Support Programme 2012–2019. Glob Health Res Policy 2020;5:13. https://doi.org/10.1186/s41256-020-00134-7.

# Additional file 2. Project documents as data sources

| Title | Author(s) | Year | Source |
| --- | --- | --- | --- |
| South-South Cooperation Assistance Fund Project Proposal: China-Africa collaboration to accelerate maternal, newborn and child health in Democratic Republic of the Congo | UNICEF | 2018 | Personal correspondence |
| China-Africa collaboration to accelerate maternal, newborn and child health in the Democratic Republic of the Congo evaluation report 2019-2021 | Tsinghua University Vanke School of Public Health | 2021 |  |
| SSCAF Project Operational Closure Report: China-Africa collaboration to accelerate maternal, newborn and child health in Democratic Republic of the Congo | UNICEF | 2022 |  |
| UNICEF Maternal, Newborn and Child Health Projects in Eight African Countries Quarterly Updates | UNICEF | 2020-2021 |  |
| South-South Cooperation Assistance Fund Training for Maternal, Newborn and Child Health Project Training Syllabus (6 November 2020-15 December 2020) | UNICEF China, IHECC | 2020 |  |
| Summative assessment table of DRC trainee’s training (in Chinese) | IHECC | 2021 |  |
| Need assessment of the eight countries for the first round training | IHECC | 2021 |  |
| Executive Summary on the South-South Cooperation Assistance Fund Training for Maternal, Newborn and Child Health Project | IHECC | 2022 |  |
| Training for China-Africa Collaboration to Improve Maternal, Newborn and Child Health in Eight African Countries | UNICEF | 2022 | UNICEF website |
